# Time to Treatment and In-Hospital Major Adverse Cardiac Events Among Patients With ST-Segment Elevation Myocardial Infarction Who Underwent Primary Percutaneous Coronary Intervention (PCI) According to the 24/7 Primary PCI Service Registry in Iran: Cross-Sectional Study

**DOI:** 10.2196/20352

**Published:** 2020-12-16

**Authors:** Younes Nozari, Babak Geraiely, Kian Alipasandi, Seyedeh Hamideh Mortazavi, Negar Omidi, Hassan Aghajani, Alireza Amirzadegan, Hamidreza Pourhoseini, Mojtaba Salarifar, Mohammad Alidoosti, Ali-Mohammad Haji-Zeinali, Ebrahim Nematipour, Mahin Nomali

**Affiliations:** 1 Department of Interventional Cardiology Tehran Heart Center Tehran University of Medical Sciences Tehran Iran; 2 Department of Cardiology School of Medicine, Tehran Heart Center Tehran University of Medical Sciences Tehran Iran; 3 Department of Cardiology Tehran Heart Center Tehran University of Medical Sciences Tehran Iran; 4 Department of Epidemiology & Biostatistics School of Public Health Tehran University of Medical Sciences Tehran Iran

**Keywords:** ST-segment elevation myocardial infarction, time to treatment, percutaneous coronary intervention, registries, Iran

## Abstract

**Background:**

Performing primary percutaneous coronary intervention (PCI) as a preferred reperfusion strategy for patients with ST-segment elevation myocardial infarction (STEMI) may be associated with major adverse cardiocerebrovascular events (MACCEs). Thus, timely primary PCI has been emphasized in order to improve outcomes. Despite guideline recommendations on trying to reduce the door-to-balloon time to <90 minutes in order to reduce mortality, less attention has been paid to other components of time to treatment, such as the symptom-to-balloon time, as an indicator of the total ischemic time, which includes the symptom-to-door time and door-to-balloon time, in terms of clinical outcomes of patients with STEMI undergoing primary PCI.

**Objective:**

We aimed to determine the association between each component of time to treatment (ie, symptom-to-door time, door-to-balloon time, and symptom-to-balloon time) and in-hospital MACCEs among patients with STEMI who underwent primary PCI.

**Methods:**

In this observational study, according to a prospective primary PCI 24/7 service registry, adult patients with STEMI who underwent primary PCI in one of six catheterization laboratories of Tehran Heart Center from November 2015 to August 2019, were studied. The primary outcome was in-hospital MACCEs, which was a composite index consisting of cardiac death, revascularization (ie, target vessel revascularization/target lesion revascularization), myocardial infarction, and stroke. It was compared at different levels of time to treatment (ie, symptom-to-door and door-to-balloon time <90 and ≥90 minutes, and symptom-to-balloon time <180 and ≥180 minutes). Data were analyzed using SPSS software version 24 (IBM Corp), with descriptive statistics, such as frequency, percentage, mean, and standard deviation, and statistical tests, such as chi-square test, *t* test, and univariate and multivariate logistic regression analyses, and with a significance level of <.05 and 95% CIs for odds ratios (ORs).

**Results:**

Data from 2823 out of 3204 patients were analyzed (mean age of 59.6 years, SD 11.6 years; 79.5% male [n=2243]; completion rate: 88.1%). Low proportions of symptom-to-door time ≤90 minutes and symptom-to-balloon time ≤180 minutes were observed among the study patients (579/2823, 20.5% and 691/2823, 24.5%, respectively). Overall, 2.4% (69/2823) of the patients experienced in-hospital MACCEs, and cardiac death (45/2823, 1.6%) was the most common cardiac outcome. In the univariate analysis, the symptom-to-balloon time predicted in-hospital MACCEs (OR 2.2, 95% CI 1.1-4.4; *P*=.03), while the symptom-to-door time (OR 1.4, 95% CI 0.7-2.6; *P*=.34) and door-to-balloon time (OR 1.1, 95% CI 0.6-1.8, *P*=.77) were not associated with in-hospital MACCEs. In the multivariate analysis, only symptom-to-balloon time ≥180 minutes was associated with in-hospital MACCEs and was a predictor of in-hospital MACCEs (OR 2.3, 95% CI 1.1-5.2; *P*=.04).

**Conclusions:**

A longer symptom-to-balloon time was the only component associated with higher in-hospital MACCEs in the present study. Efforts should be made to shorten the symptom-to-balloon time in order to improve in-hospital MACCEs.

**International Registered Report Identifier (IRRID):**

RR2-10.2196/13161

## Introduction

Acute myocardial infarction with and without ST-segment elevation is a prevalent cardiac emergency, which is responsible for potential morbidity and mortality worldwide [1]. For patients with ST-segment elevation myocardial infarction (STEMI), primary percutaneous coronary intervention (PCI) has been considered as the preferred reperfusion therapy, which can be provided by an experienced team [2]. Additionally, for patients with contraindication of thrombolytic therapy, this reperfusion strategy can be a reliable substitute [3]. Moreover, in high-volume hospitals, primary PCI can be performed faster and can lead to lower mortality [4]. However, performing primary PCI for patients with STEMI may be associated with major adverse cardiocerebrovascular events (MACCEs) [5]. On the other hand, introducing a STEMI network, such as a 24/7 primary PCI regional service, may lead to improved accessibility for invasive diagnosis and treatment and may reduce mortality [6].

Although a recent guideline emphasized a timely reperfusion strategy in patients with STEMI [7], a longer time to treatment has been found [8]. According to Kim et al, the time to treatment has different components, including the symptom-to-door time (ie, time from symptom onset to hospital arrival), door-to-balloon time (ie, time from hospital arrival to balloon inflation), and symptom-to-balloon time (ie, time from symptom onset to balloon inflation) [9]. A longer time to treatment may affect clinical outcomes following primary PCI [10]. Thus, any delay related to time to treatment should be noticed [2] and should be recorded and reviewed regularly in every system providing care to patients with STEMI [11].

Although several studies reported no improvement in clinical outcomes and survival of patients who underwent primary PCI, despite improvement in the door-to-balloon time over the years [9,12-15], other studies focused on the door-to-balloon time because of an association between a lower door-to-balloon time and better outcomes in terms of both in-hospital outcomes and long-term survival [16-21]. On the other hand, other components of time to treatment, such as the symptom-to-balloon time and symptom-to-door time, have not been considered. The symptom-to-balloon time, as an estimate of total ischemic time, is strongly correlated with infarct size and mortality compared with its subintervals, such as the door-to-balloon time and symptom-to-door time, as a substantial duration of myocardial ischemia prior to hospital arrival accounts for a large number of deaths during the prehospital period [22]. Therefore, it is required to pay attention to all components of time to treatment when evaluating MACCEs among patients with STEMI who have undergone primary PCI.

According to a research project in Iran (Iranian Project for Assessment of Coronary Events 2 [IPACE2]), despite relatively timely in-hospital reperfusion performed for patients with STEMI, long-time patient delay was found [23]. However, its impact on MACCEs among patients with STEMI undergoing primary PCI in a health care setting of a developing country, such as Iran, has not been evaluated. On the other hand, previous studies indicated controversy among different components of treatment times and short-term and long-term MACCEs [13,19,24,25]. Therefore, owing to the predictive role of treatment times in clinical outcomes, the association between time to treatment and 1-month mortality was studied by Kim et al in 2017 for the first time [9]. However, this association with in-hospital MACCEs has not been evaluated. In addition, previous studies have been performed in developed countries, which may limit applicability in Iran having a different quality of care.

There is a lack of information on the association between time to treatment and in-hospital MACCEs among patients with STEMI undergoing primary PCI in Iran, and such information can help health care systems to identify sources of time delays, achieve better planning, and apply preventive strategies for improving clinical outcomes following primary PCI in order to plan and manage STEMI more properly. Thus, we designed and conducted this study to determine the association between all components of time to treatment (ie, symptom-to-door time, door-to-balloon time, and symptom-to-balloon time) and in-hospital MACCEs among patients with STEMI who underwent primary PCI, according to the 24/7 primary PCI service registry of Tehran Heart Center (THC) in Iran.

## Methods

### Study Design and Setting

We conducted an observational study according to the 24/7 primary PCI service registry of THC in Iran. Details of the study design and setting have been published previously [26]. The study protocol was approved by the institutional review board and research ethics committee of Tehran University of Medical Sciences. The Strengthening the Reporting of Observational Studies in Epidemiology (STROBE) guidelines were used to report study results [27].

### Participants

All patients who were treated with primary PCI at one of the six catheterization laboratories of THC between November 2015 and August 2019, and whose data were registered prospectively in the 24/7 primary PCI service registry were included as study participants.

Inclusion criteria for this study were age older than 18 years, confirmed diagnosis of STEMI, and primary PCI through a standard technique without bolus administration of fibrinolytic agents in the catheterization laboratory of THC. Those with incomplete data regarding one of the time to treatment variables (eg, symptom-to-door time, door-to-balloon time, and symptom-to-balloon time) and the study outcome (ie, in-hospital MACCEs) were excluded from the analysis [26].

### Data Sources

We used the registered data in the 24/7 primary PCI service registry as the main source of data. Details of the registry have been previously published [26]. Collected data from the time of admission to the emergency department (ED) to transfer to the catheterization laboratory through the STEMI management registry form were entered in the 24/7 primary PCI service registry by research staff weekly [26]. A flow chart of patient admission to the ED and transfer to the catheterization laboratory of THC has been published previously [28].

### Variables

We explained study variables in the study protocol completely [26]. Demographic and clinical variables were recorded. Demographic variables were age, sex, BMI, and current smoking. Clinical variables were medical history of myocardial infarction (MI), PCI, and coronary artery bypass grafting (CABG); comorbidities of diabetes, hypertension, and hyperlipidemia; family history of cardiovascular diseases (CVDs); previous cardiopulmonary resuscitation (CPR); emergency medical service (EMS) user; infarcted territories including anterior, posterior, inferior, or lateral; infarct-related arteries (IRAs) including the graft, left main, left anterior descending, left circumflex, and right coronary arteries; multivessel disease; procedural support including pacemaker, mechanical ventilation, intra-aortic balloon pump, inotropes, cardioversion, and defibrillator; and pre- and postprimary PCI thrombolysis in myocardial infarction (TIMI) flow.

The main independent variables were time to treatment, including the symptom-to-door time (ie, time from self-reported onset of symptoms to hospital arrival), door-to-balloon time (ie, time from hospital arrival to reperfusion), and symptom-to-balloon time (time from self-reported onset of symptoms to reperfusion). All components of time to treatment were recorded as continuous quantitative variables (in hours and minutes), and then, they were reported separately (in minutes) as categorical variables (ie, symptom-to-door time and door-to-balloon time <90 and ≥90 minutes; symptom-to-balloon time <180 and ≥180 minutes), according to the study by Kim et al [9]. The study outcome was in-hospital MACCEs, which were measured before hospital discharge. In-hospital MACCEs represented a composite index including different elements of MI, stroke, cardiac death, target vessel revascularization, and target lesion revascularization that have been defined previously [26]. It should be noted that first medical contact and STEMI diagnosis have been considered according to the definition provided in the 2017 European Society of Cardiology guidelines [11]. Therefore, first medical contact time was the time between hospital arrival and initial assessment by a physician, paramedic, or nurse, or other trained EMS personnel who could obtain and interpret an electrocardiogram (ECG). Additionally, STEMI diagnosis time was the time between first medical contact to STEMI ECG verification and diagnosis, which was reported categorically (ie, ≤10 minutes and >10 minutes) [11].

### Statistical Methods

We compared the baseline characteristics of patients who underwent primary PCI across in-hospital MACCEs. Continuous variables are reported as mean (SD) or median (IQR) and were compared using independent sample *t* tests or Mann-Whitney *U* tests. In order to compare categorical variables, chi-square tests were used, and the data are reported as proportions and percentages.

Univariate binary logistic regression analysis was performed to determine the relationship between time to treatment and in-hospital MACCEs and between other variables and in-hospital MACCEs. In order to modify the effect of other dependent variables on the studied relationship, variables with a significance level of ≤.2 in the univariate binary logistic regression were taken for multivariate analysis and entered in a multiple logistic regression model. After removing insignificant variables through backward elimination regression analysis, the studied relationship was reported.

Each relationship between the variables was expressed as an odds ratio (OR) with a 95% CI. All statistical tests were set as two-tailed tests. A *P* value <.05 was considered statistically significant. The statistical package IBM SPSS for Windows, version 24.0 (IBM Corp) was used for the statistical analyses.

## Results

### Participants

**Figure 1:**
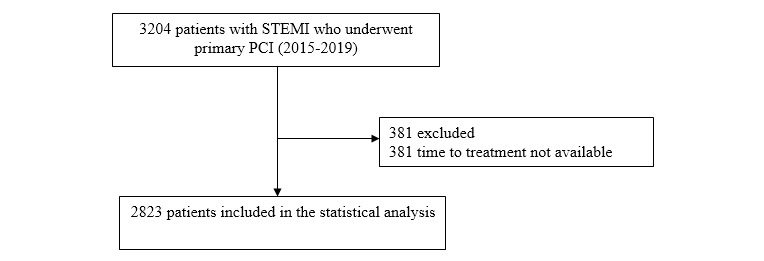
Study population diagram. PCI: percutaneous coronary intervention; STEMI: ST-segment elevation myocardial infarction.

From November 2015 to August 2019, 3204 consecutive patients with STEMI underwent primary PCI in THC, and their data were recorded in the 24/7 primary PCI service registry. Patients were excluded if time to treatment data were not available. Thus, 2823 out of 3204 patients were included in this study (completion rate: 88.1%) (Figure 1).

### Descriptive Data

Baseline characteristics of the study patients are shown in Table 1. According to Table 1, the mean age of the study population was 59.6 years, with overall obese BMI. There were high proportions of male sex and hyperlipidemia and low proportions of current smoking, EMS use, family history of CVDs, previous CPR, and medical history of MI, PCI, and CABG among the study population (Table 1).

**Table 1:** Baseline characteristics of the study patients and comparison according to in-hospital major adverse cardiocerebrovascular events.

Characteristic			Overall value	In-hospital MACCEs^a^, value		*P* value
				Yes	No	
**Demographic characteristics**						
	Age (years) (n=2823), mean (SD)		59.6 (11.6)	65.8 (12.6)	59.5 (11.6)	<.001^b^
	Age ≥75 years, n/N (%)		316/2823 (11.2)	18 (26.1)	298 (10.8)	
	**Sex**					
		Male, n/N (%)	2243/2823 (79.5)	47/69 (68.1)	2196/2754 (79.7)	.02^b^
		Female, n/N (%)	580/2823 (20.5)	22/69 (31.9)	558/2754 (20.3)	
	BMI (kg/m^2^) (n=2760), mean (SD)		27.8 (4.4)	27.9 (4.3)	27.8 (4.4)	.80
	Current smoker, n/N (%)		1045/2803 (37.3)	14/68 (20.6)	1031/2735 (37.7)	.004^b^
**Clinical characteristics**						
	EMS^c^ user, n/N (%)		456/2463 (18.5)	9/54 (16.7)	447/2409 (18.6)	.72
	Family history of CVDs^d^, n/N (%)		445/2749 (16.2)	10/68 (14.7)	435/2681 (16.2)	.74
	Previous CPR^e^, n/N (%)		62/2755 (2.3)	4/68 (5.9)	58/2687 (2.2)	.04^b^
	**Medical history**					
		MI^f^, n/N (%)	350/2755 (12.7)	7/68 (10.3)	343/2687 (12.8)	.55
		PCI^g^, n/N (%)	424/2755 (15.4)	5/68 (7.4)	419/2687 (15.6)	.06
		CABG^h^, n/N (%)	132/2755 (4.8)	2/68 (2.9)	130/2687 (4.8)	.47
	**Comorbidities**					
		Diabetes mellitus, n/N (%)	1135/2803 (40.5)	33/68 (48.5)	1102/2735 (40.3)	.17
		Hypertension, n/N (%)	1307/2803 (46.6)	35/68 (51.5)	1272/2735 (46.5)	.42
		Hyperlipidemia, n/N (%)	1493/2803 (53.3)	28/68 (41.2)	14.65/2735 (53.6)	.04^b^
	FMC^i^ (min) (n=2823), median (IQR)		1 (0-6)	2 (0-7)	1 (0-6)	.33
	STEMI^j^ diagnosis time ≤10 min, n/N (%)		1923/2823 (68.4)	45/69 (65.2)	1885/2754 (68.4)	.57
	Symptom-to-door time <90 min, n/N (%)		579/2823 (20.5)	11/69 (15.9)	568/2754 (20.6)	.34
	Door-to-balloon time <90 min, n/N (%)		2089/2823 (74.0)	50/69 (72.5)	2039/2754 (74.0)	.77
	Symptom-to-balloon time <180 min, n/N (%)		691/2823 (24.5)	9/69 (13.0)	682/2754 (24.8)	.03^b^
	**Infarct-related artery**					
		Graft, n/N (%)	91/2823 (3.2)	2/69 (2.9)	89/2754 (3.2)	.88
		Left main, n/N (%)	21/2823 (0.7)	3/69 (4.3)	18/2754 (0.7)	<.001^b^
		Left anterior descending, n/N (%)	1377/2823 (48.8)	45/69 (65.2)	1332/2754 (48.4)	.006^b^
		Left circumflex, n/N (%)	668/2823 (23.7)	13/69 (18.8)	655/2754 (23.8)	.34
		Right coronary, n/N (%)	965/2823 (34.2)	22/69 (31.9)	943/2754 (34.2)	.68
	Preprimary PCI TIMI^k^ flow <3, n/N (%)		2249/2499 (90.0)	60/65 (92.3)	2189/2434 (89.9)	.53
	Postprimary PCI TIMI flow <3, n/N (%)		206/2493 (8.3)	15/65 (23.1)	191/2428 (7.9)	<.001^b^
	**Infarcted territory**					
		Anterior, n/N (%)	1286/2823 (45.6)	43/69 (62.3)	1243/2754 (45.1)	.005^b^
		Inferior, n/N (%)	1240/2823 (43.9)	24/69 (34.8)	1216/2754 (44.2)	.12
		Lateral, n/N (%)	425/2823 (15.1)	12/69 (17.4)	413/2754 (15.0)	.58
		Posterior, n/N (%)	302/2823 (10.7)	4/69 (5.8)	298/2754 (10.8)	.18
	Multivessel disease, n/N (%)		1836/2773 (66.2)	48/69 (69.6)	1788/2704 (66.1)	.55
	Procedural supports, n/N (%)		29/2820 (1.0)	9/69 (13.0)	20/2751 (0.7)	<.001^b^

^a^MACCEs: major adverse cardiocerebrovascular events.

^b^*P* value for independent sample *t* tests and chi-square tests.

^c^EMS: emergency medical service.

^d^CVDs: cardiovascular diseases.

^e^CPR: cardiopulmonary resuscitation.

^f^MI: myocardial infarction.

^g^PCI: percutaneous coronary intervention.

^h^CABG: coronary artery bypass graft surgery.

^i^FMC: first medical contact.

^j^STEMI: ST-segment elevation myocardial infarction.

^k^TIMI: thrombolysis in myocardial infarction.

The study patients were initially assessed by the medical team with a median time of 1 minute (first medical contact time of 1 minute). For the majority of patients, STEMI was diagnosed in less than 10 minutes after obtaining the first ECG. The median door-to-balloon time was 55 (IQR 40-92) minutes, and door-to-balloon time ≤90 minutes was noted in most of the patients. The median symptom-to-door time and symptom-to-balloon time were 258 (IQR 108-574) and 355 (180-720) minutes, respectively. Additionally, low proportions of symptom-to-door time ≤90 minutes and symptom-to-balloon time ≤180 minutes were observed among the study patients. The left anterior descending artery was the most common infarct-related artery, and the anterior infarcted territory was seen in the majority of patients. Most of the patients had preprimary PCI TIMI flow <3, and a low proportion of patients had postprimary PCI TIMI flow <3. Multivessel disease was observed in the majority of patients, and only 1.03% (29/2820) of patients received procedural support (Table 1).

### Outcome Data

During the study period, in-hospital MACCEs occurred in 69 patients (N=2823, 2.4%), and cardiac death (45/2823, 1.6%), target vessel revascularization/target lesion revascularization (16/2823, 0.57%), MI (5/2823, 0.18%), and stroke (3/2823, 0.11%) were the most common events.

Baseline characteristics according to in-hospital MACCEs are presented in Table 1. In the in-hospital MACCE group, there were more women and patients with age ≥75 years, previous CPR, left main and left anterior descending arteries as IRAs, anterior infarcted territory, initial TIMI flow <3, and procedural support. In contrast, less patients with current smoking, hyperlipidemia, and final TIMI flow <3 were seen in the in-hospital MACCE group (Table 1).

From the different components of time to treatment, only symptom-to-balloon time ≥180 minutes was significantly higher in the in-hospital MACCE group (*P*=.03). Other characteristics were similar between the two groups (Table 1).

### Main Results

In the univariate analysis, the symptom-to-balloon time predicted in-hospital MACCEs (OR 2.2, 95% CI 1.1-4.4; *P*=.03), while the symptom-to-door time (OR 1.4, 95% CI 0.7-2.6; *P*=.34) and door-to-balloon time (OR 1.1, 95% CI 0.6-1.8; *P*=.77) were not associated with in-hospital MACCEs.

In the multivariate analysis, after adjustment by age ≥75 years; female gender; current smoking; diabetes mellitus and hyperlipidemia; history of CPR; medical history of PCI; left main and left anterior descending arteries as IRAs; final TIMI flow <3; procedural support; and anterior, inferior, and posterior territories of MI (Multimedia Appendix 1), only symptom-to-balloon time ≥180 minutes was associated with in-hospital MACCEs and was a predictor of in-hospital MACCEs (OR 2.3, 95% CI 1.1-5.2; *P*=.04) (Table 2).

**Table 2:** Univariate and multivariate analyses of time to treatment and in-hospital major adverse cardiocerebrovascular events.

Characteristic	Unadjusted OR^a^ (95% CI)	*P* value	Adjusted^b^ OR (95% CI)	*P* value
Symptom-to-door time ≥90 min	1.4 (0.7-2.6)	.34	N/A^c^	N/A
Door-to-balloon time ≥90 min	1.1 (0.6-1.8)	.77	N/A	N/A
Symptom-to-balloon time ≥180 min	2.2 (1.1-4.4)	.03^d^	2.3 (1.1-5.2)	.04^e^

^a^OR: odds ratio.

^b^Adjusted by age ≥75 years; female gender; current smoking; diabetes mellitus and hyperlipidemia; history of cardiopulmonary resuscitation; medical history of percutaneous coronary intervention; left main and left anterior descending arteries as infarct-related arteries; final thrombolysis in myocardial infarction flow <3; procedural support; and anterior, inferior, and posterior territory of myocardial infarction.

^c^N/A: not applicable.

^d^Included in the multiple logistic regression model (*P*<.2).

^e^*P* value <.05.

## Discussion

To our knowledge, this is the first study to evaluate the association of treatment times and in-hospital MACCEs among patients with STEMI who underwent primary PCI, according to a 24/7 primary PCI service registry in Iran.

Our study findings indicated that the majority of patients had symptom-to-balloon time ≥180 minutes, and it was the only component of time to treatment that was an independent predictor of in-hospital MACCEs among study patients. However, in the study by Song et al in China, there was no association between longer symptom-to-balloon time and in-hospital mortality or MACCEs [25], which may be due to the different classification of the symptom-to-balloon time. In addition, regarding the relationship between treatment time and short-term outcomes, the study by Kim et al showed that a total ischemic time <180 minutes could be a predictor of 1-month mortality and could lead to a relevant reduction in the 1-month mortality incidence compared with symptom-to-balloon time ≥180 minutes [9]. Moreover, symptom-to-balloon time ≤240 minutes was reported as a strong predictor of 1-year major adverse cardiovascular events (MACEs) [24]. What is clear in previous studies is the different classifications of the symptom-to-balloon time, which makes it difficult to compare the results of studies and provide a single conclusion about the association between the symptom-to-balloon time and clinical outcomes. Although the door-to-balloon time is well known as a clinical indicator of care quality [29], the symptom-to-balloon time is an estimate of total ischemic time, which is strongly correlated with infarct size and mortality [22], and a longer symptom-to-balloon time results in impaired myocardial perfusion [25] and worse ejection fraction when the left anterior descending artery is the culprit vessel [30]. Thus, the symptom-to-balloon time can be the correct focus of attention for optimal STEMI care instead of its subintervals, such as the door-to-balloon time [22]. In addition, owing to the higher frequency of patients with a longer symptom-to-balloon time in our study setting, efforts and planning should be focused on improving prehospital STEMI diagnosis and direct transfer to the catheterization laboratory, which shortens the treatment time compared with diagnosis in the ED, and it could be associated with less mortality [31].

The symptom-to-balloon time is a combination of two subintervals, including the symptom-to-door time and door-to-balloon time. In our study, the majority of patients had a shorter door-to-balloon time, and the door-to-balloon time was not an independent predictor of in-hospital MACCEs, which was consistent with the finding in the study by Kim et al [9]. In contrast to our study, it has been shown that shortening the door-to-balloon time, even if less than 60 to 90 minutes, is associated with survival benefits [20,32] and lower mortality over time [21]. Moreover, according to a systematic review and meta-analysis in 2019, a longer door-to-balloon time (≥90 minutes) is associated with a higher risk of mortality [33]. A door-to-balloon time of <90 minutes depends on hospital systems and can be achieved easily with effective hospital strategies [34] and by emphasizing on guideline adherence in order to minimize reperfusion delay and improve survival among patients with STEMI undergoing primary PCI [20].

Another subinterval of the symptom-to-balloon time is the symptom-to-door time. In this study, a longer symptom-to-door time was seen, and it was not associated with in-hospital MACCEs. According to a previous study in THC, a higher symptom-to-door time was associated with female gender, transfer via vehicles other than an ambulance, atypical chest pain, low level of education, late night and morning onset of pain, history of hypertension, and opium abuse, whereas a history of CABG was associated with lower prehospital delay [35]. In addition, it was negatively associated with postinfarction left ventricular ejection fraction in patients with STEMI [36]. Although a door-to-balloon time target of <90 minutes can be achieved easily by effective hospital strategies [34], the time taken for the patient to recognize ischemic symptoms is the main contributor to a longer total ischemic time [34]. Thus, public education about cardiovascular symptoms and a prompt emergency call is necessary in order to reduce the symptom-to-door time in patients with STEMI [37].

In conclusion, among the different components of time to treatment, the symptom-to-balloon time was the only component that was associated with in-hospital MACCEs in the study patients, and a longer symptom-to-balloon time was associated with higher in-hospital MACCEs. It seems that attention should be shifted from the door-to-balloon time, as a care quality indicator among primary PCI service providers, to the symptom-to-balloon time, as the total ischemic duration, in order to improve clinical outcomes in patients with STEMI undergoing primary PCI. In order to shorten the symptom-to-balloon time and improve clinical outcomes, prehospital emergency systems should be improved and the symptom-to-door time, as the main contributor to a longer symptom-to-balloon time, should be improved by special educational programs to raise public awareness on STEMI symptoms and prompt seeking of medical care.

There were several limitations in this study. First, it was a single-center observational study. Thus, no causal association between time to treatment and MACCEs could be proven conclusively. Second, the study population included patients with STEMI treated with primary PCI, and the study findings cannot be applied to patients with STEMI receiving thrombolytic therapy. Third, Killip class is a variable that may have affected the study results. However, because it was not recorded in the registry, we did not consider it in the analysis.

## Appendix

Multimedia Appendix 1 Univariate and multivariate analyses of time to treatment and in-hospital major adverse cardiocerebrovascular events.

## Abbreviations

CABG: coronary artery bypass grafting
CPR: cardiopulmonary resuscitation
CVDs: cardiovascular diseases
ECG: electrocardiogram
ED: emergency department
EMS: emergency medical service
IRA: infarct-related artery
MACCEs: major adverse cardiocerebrovascular events
MI: myocardial infarction
OR: odds ratio
PCI: percutaneous coronary intervention
STEMI: ST-segment elevation myocardial infarction
THC: Tehran Heart Center
TIMI: thrombolysis in myocardial infarction
