# Supplementary material for: Time to Treatment and In-Hospital Major Adverse Cardiac Events Among Patients With ST-Segment Elevation Myocardial Infarction Who Underwent Primary Percutaneous Coronary Intervention (PCI) According to the 24/7 Primary PCI Service Registry in Iran: Cross-Sectional Study
Source: Interact J Med Res. 2020 Dec 16;9(4):e20352. doi: 10.2196/20352 (PMC7773509; doi:10.2196/20352)
Supplement: Multimedia Appendix 1 [file ijmr_v9i4e20352_app1.docx]

Appendix1:

Table2a. Univariate and multivariate analysis of time-to-treatment and in-hospital MACCEs

| Characteristics | | | Unadjusted OR^[[1]](#footnote-1)^  (95% CI^[[2]](#footnote-2)^) | P-value | Adjusted OR (95% CI) | P-value |
| --- | --- | --- | --- | --- | --- | --- |
|  | Symptom-to-door time≥90 min | | 1.4 (0.7-2.6) | 0.343 |  |  |
|  | Door-to-balloon time ≥90 min | | 1.1 (0.6-1.8) | 0.769 |  |  |
|  | Symptom-to-balloon time ≥180 min | | 2.2 (1.1-4.4) | 0.029* | 2.3 (1.1-5.2) | 0.041** |
|  | Age≥75 | | 2.9 (1.7-5.1) | <0.001* | 2.4 (1.3-4.5) | 0.004** |
|  | Female sex | | 1.8 (1.1-3.1) | 0.020* |  |  |
|  | BMI^[[3]](#footnote-3)^ (kg/m^2^) | | 1.1 (0.9-1.1) | 0.803 |  |  |
|  | Current smoker | | 0.4 (0.2-0.7) | 0.005* | 0.4 (0.2-0.8) | 0.016** |
|  | EMS ^[[4]](#footnote-4)^ non user | | 1.2 (0.5-2.3) | 0.724 |  |  |
|  | Family history of CVDs^[[5]](#footnote-5)^ | | 0.9 (0.4-1.7) | 0.737 |  |  |
|  | Previous CPR^[[6]](#footnote-6)^ | | 2.8 (0.9-8.1) | 0.050* | 3.3 (1.1-10.7) | 0.045** |
|  | Past medical history | |  |  |  |  |
|  |  | MI^[[7]](#footnote-7)^ | 0.7 (0.3-1.7) | 0.547 |  |  |
|  |  | PCI^[[8]](#footnote-8)^ | 0.4 (0.1-1.1) | 0.071* | 0.4 (0.1-1.1) | 0.054 |
|  |  | CABG^[[9]](#footnote-9)^ | 0.6 (0.2-2.5) | 0.474 |  |  |
|  | Comorbidities | |  |  |  |  |
|  |  | Diabetes mellitus | 1.4 (0.8-2.3) | 0.174 |  |  |
|  |  | Hypertension | 1.2 (0.7-1.9) | 0.418 |  |  |
|  |  | Hyperlipidemia | 0.6 (0.4-0.9) | 0.045* |  |  |
|  | Infarct-related artery | |  |  |  |  |
|  |  | Graft | 0.8 (0.2-3.7) | 0.877 |  |  |
|  |  | Left main | 6.9 (1.9-24.0) | 0.002* | 6.4 (1.3-28.9) | 0.024** |
|  |  | Left anterior descending | 2.1 (1.2-3.3) | 0.007* | 2.1 (1.2-3.5) | 0.010** |
|  |  | Left circumflex | 0.7 (0.4-1.4) | 0.342 |  |  |
|  |  | Right coronary | 0.9 (0.5-1.5) | 0.684 |  |  |
|  | Preprimary PCI TIMI^g^ flow <3 | | 1.3 (0.5-3.4) | 0.531 |  |  |
|  | Post primary PCI TIMI flow <3 | | 3.5 (1.9-6.4) | <0.001* | 2.7 (1.4-5.3) | 0.002** |
|  | Infarcted territory | |  |  |  |  |
|  |  | Anterior | 2.1 (1.2-3.3) | 0.005* |  |  |
|  |  | Inferior | 0.6 (0.4-1.1) | 0.124* |  |  |
|  |  | Lateral | 1.2 (0.6-2.2) | 0.583 |  |  |
|  |  | Posterior | 0.5 (0.2-1.4) | 0.191* |  |  |
|  | Multi-vessel disease | | 1.2 (0.7-1.9) | 0.551 |  |  |
|  | Procedural supports | | 20.4 (8.9-46.8) | <0.001* | 13.3 (5.2-34.4) | <0.001** |

* Included in the multiple logistic regression model (P value <0.2)

**P value less than 0.05

1. OR: Odds ratio [↑](#footnote-ref-1)
2. CI: Confidence interval [↑](#footnote-ref-2)
3. BMI: Body mas index [↑](#footnote-ref-3)
4. EMS: Emergency medical service [↑](#footnote-ref-4)
5. CVDs: Cardiovascular diseases [↑](#footnote-ref-5)
6. CPR: Cardiopulmonary resucitation [↑](#footnote-ref-6)
7. MI: Myocardial infarction [↑](#footnote-ref-7)
8. PCI: Percutaneous coronary intervention [↑](#footnote-ref-8)
9. CABG: Coronary artery bypass graft surgery [↑](#footnote-ref-9)
